# Two Recent Cases of Babesiosis in the United States With a Difference in Severity and Response to Therapy

**DOI:** 10.7759/cureus.6853

**Published:** 2020-02-03

**Authors:** Ulviyya Gasimova, Ganapathiram Arivudainambi, Sam Joseph, Salaheldin Elhamamsy

**Affiliations:** 1 Internal Medicine, Nardone Medical Associates, Pawtucket, USA; 2 Internal Medicine, University of Massachusetts Medical School, Boston, USA; 3 Internal Medicine, Saint Mary Medical Center, Langhorne, USA; 4 Internal Medicine, Warren Alpert Brown Medical School, Providence, USA

**Keywords:** babesiosis, hemolytic anemia, b. microti, tick-borne infections

## Abstract

Being one of the widespread parasitic infections in particular parts of the United States, babesiosis may present with varying severity of clinical manifestations. Depending on the severity and degree of hemolysis, some patients may need a more aggressive approach, such as repeated red blood cell (RBC) transfusions, while in others, symptoms may be well-controlled with conservative therapy only using a disease-specific approach. We are presenting two cases with a significant difference in severity and medical therapy.

## Introduction

Babesiosis is a parasitic infectious disease caused by one of four Babesia species [1-2]. Babesia microti (B. microti) is the most common species in the United States and is vectored by Ixodes scapularis during the months of May to October [3-4]. As widespread as Lyme disease in some parts of the country, babesiosis can present with high fevers (reaching >105F), fatigue, malaise, headache, myalgia, and arthralgia [4-6]. Hemolytic anemia and thrombocytopenia are also hallmarks of the disease [7]. We present two recent cases of babesiosis in comparable patients with vastly different severity of clinical presentation and response to clinical management.

Babesiosis is an intraerythrocytic protozoan infection caused by the genus Babesia. Endemic to the northeastern and upper midwestern United States, B. microti is most often associated with human infection. The life cycle of the parasite is carried through in the white-footed mice and Ixodes scapularis ticks that feed on rodents. Humans are incidental hosts and become infected following a bite from an infected tick. Ixodes scapularis is also known to vector Borrelia burgdorferi (B. burgdorferi), and Anaplasma phagocytophilum; therefore, coinfection is common in babesiosis [8]. The presentation of the disease can range from asymptomatic to severe, life-threatening hemolytic anemia in immunocompromised individuals [4,7,9-11]. The treatment of symptomatic patients includes antibiotics, antimalarial drugs, and red blood cell (RBC) exchange transfusions [12].

## Case presentation

We present two recent babesiosis cases in the New England area of the United States. Although both patients are of similar age, have similar risk factors and reported tick bites in similar geographical areas, the severity of their clinical presentation and response to therapy were markedly different.

**Figure 1:**
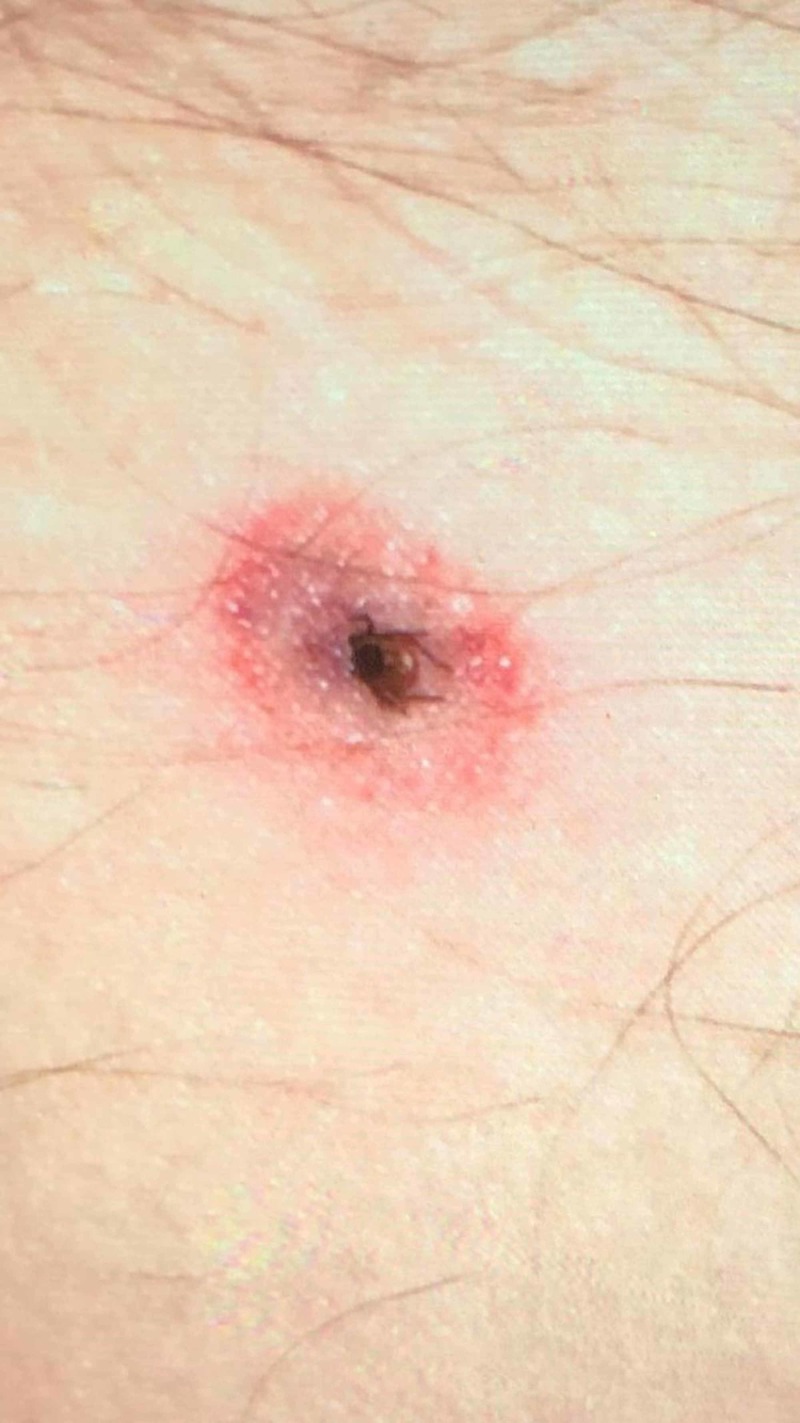
Ixodes scapularis tick attached to the anterior abdominal wall

Case 1 is a 51 years-old male with no significant past medical history who presented to the emergency room on June 29, 2019, with the history of a tick bite on the anterior abdominal wall four months prior (Figure 1).

On presentation, the patient complained of generalized weakness, fatigue, myalgia, lightheadedness, and achiness that had started approximately a week ago. During this period, he reported intermittent, subjective fevers. He also began experiencing intermittent headaches, which were more prominent on the right posterior side, with seven out of 10 in severity. Four days prior to presentation, the patient had visited his primary care physician with these complaints, and a complete blood count (CBC), comprehensive metabolic panel, and tick-borne panel were ordered and metaxalone (muscle relaxant) was prescribed. His symptoms did not improve on this regimen and he developed dark urine two days prior to presentation.

On presentation to the emergency room, the patient was found to have temperature 103.2 F, blood pressure (BP) 142/94, pulse 122, respiratory rate (RR) 18, and oxygen saturation 94% to 95%. The remainder of the examination was unremarkable. CBC showed white blood cells (WBCs) 8.4 103/uL, hemoglobin 14.4 g/dL, hematocrit 40.6%, and platelet count 51,103/uL. Comprehensive metabolic panel (CMP) revealed an increase in total bilirubin 3.7 mg/dL, with direct bilirubin 1.02 mg/dL and an increase in liver enzymes. The results of CBC and CMP ordered by his primary care physician showed similar results. The tick-borne panel from the visit showed a negative Anaplasma polymerase chain reaction (PCR), negative Lyme disease serology, and positive Babesia PCR. The patient was admitted for further workup and management.

On admission, the patient was clinically and hemodynamically stable with an unclear degree of parasitemia. The patient was started on a regimen of azithromycin 500 mg four times a day in addition to atovaquone 750 mg two times a day. The patient was closely monitored for improvement of his symptoms along with daily CBC, CMP, and Babesia parasitemia. On hospital day one, the Babesia parasitemia level was 12.1%. The patient was hemodynamically stable and liver functions were improving on the treatment regimen. On hospital day two, the patient’s hemoglobin dropped from 14.5 g/dL to 12 g/dL. Blood smear showed a parasitemia of 13%. Hematology and Infectious Disease (ID) were consulted. On hospital day three, the patient reported feeling increasingly unwell and developed shortness of breath. Daily lab results were remarkable for worsening thrombocytopenia and a blood parasite load of 42%. Given increasing parasitemia despite therapy, exchange transfusion was recommended and intravenous clindamycin 900 mg every eight hours was added. On hospital day four, the patient received red blood cell (RBC) exchange transfusion, after which the parasitemia level decreased to 9.6%. Over hospital days from the fifth to the eighth days, the patient’s parasitemia decreased to 6.6%; however, the patient continued to be intermittently febrile (as high as 103.2F) managed with acetaminophen. During these febrile episodes, the patient reported difficulty breathing and would desaturate to the high 70s to low 80s, requiring intermittent supplemental oxygen (up to 3L on the venti mask). Due to the lack of satisfactory clinical improvement, doxycycline 100 mg per oral, twice a day was added from hospital days six to eight for possible tick-borne coinfection until repeat tick panel confirmed no coinfection. On hospital day eight, another exchange transfusion was done, after which parasitemia decreased to 2.1% on hospital day nine and the patient became afebrile. The patient was discharged on hospital day 10 with a parasite load of 0.1%. He was instructed to take azithromycin 500 mg per oral four times a day and atovaquone 250 mg per oral twice a day for an additional 14 days. Workup for a possible immunocompromised state during his hospitalization revealed normal spleen on left upper quadrant ultrasound imaging, negative human immunodeficiency virus (HIV) screen, and unremarkable serum protein electrophoresis (SPEP).

Case 2 was a 48-year-old male with a previous history of diabetes mellitus type II and hyperlipidemia, who presented to the emergency room on June 25, 2019, with persistent fevers. The patient had a tick bite one month prior to presentation on his posterior thigh, which was removed after two days. Two days prior to presentation, the patient developed high spiking fevers of up to 105F and visited urgent care where he was prescribed doxycycline 100 mg every 12 hours. Despite this regimen, the patient continued to be febrile and presented to the emergency room.

On presentation, the patient was febrile to 102.9F and reported fatigue, sweats, and chills. He denied experiencing headaches, myalgias, arthralgias, back pain, or other localizing symptoms. Vital signs showed pulse 130, RR 22, and BP 118/67. Blood work revealed hemoglobin 12.1g/L, hematocrit 37.1%, platelet count 77,103/uL, and an increase in liver enzymes and total bilirubin levels. The tick-borne panel showed negative Anaplasma PCR and Lyme disease serology. Blood smear showed Babesia parasites, with a parasitemia level of 4%, and infection was also later confirmed by the tick panel. The patient was started on the treatment regimen of azithromycin 500 mg intravenous daily (subsequently transitioned to per oral after four days), atovaquone 750 mg per oral twice a day, and doxycycline 100 mg per oral every 12 hours (discontinued after three days). Daily CBC, CMP, and parasitemia levels were closely followed. On hospital day two, the patient’s blood smear yielded a parasitemia level of 1.95 %, and CBC showed a drop in hemoglobin level from 12 g/L to 8.8 g/L. The patient had a temperature of 102.5F, sweating, and rigors. The patient was given intravenous (IV) fluids, acetaminophen, and ibuprofen with significant symptomatic improvement. The patient’s parasitemia level continued to decrease, and he was discharged on hospital day five. The patient was prescribed a regimen of azithromycin 500 mg per oral daily and atovaquone 750 mg twice a day for an additional six days.

## Discussion

As mentioned earlier, babesiosis in humans is a disease caused by B. microti, which uses Ixodes scapularis as a vector. Babesiosis is widespread in some parts of the United States and is endemic in New England. The incidence of the cases is the highest in the spring-to-fall season [3-4]. The disease can present with a spectrum of severity after an incubation period of one to four weeks (4). Many patients are asymptomatic. Some can present with mild to moderate disease (associated with parasitemia > 4%) and usually complain of high spiking fevers, chills, sweating, and fatigue. There can also be more prominent system involvement, which can present as myalgia, arthralgia, headache, lightheadedness, nausea, and vomiting. CBC reveals hemolytic anemia, which can be presented as a mild to moderate decrease in hemoglobin, hematocrit, and haptoglobin levels, as well as an increase in total bilirubin, direct bilirubin, and lactate dehydrogenase. One of the most prominent findings is a decrease in platelet count [6-7,13]. Severe disease is commonly associated with a parasite load of >4% and presents similarly to mild/moderate disease but with greater severity. Nausea and vomiting are more indicative of severe disease. Risk factors for severe disease in adults include age greater than 50, an immunocompromised state, and asplenia [7]. Confirmation of the diagnosis done through the identification of the parasite on thin blood smear using Giemsa or Wright staining [2,14-15]. Since Babesiosis shares the same tick vector with Lyme disease and anaplasmosis, patients suspected with any of them should be evaluated for all three using the tick-borne panel.

Both patients presented following tick bites one and four months prior, with fever as the primary complaint. However, the first presented with other associated symptoms such as fatigue, myalgias, and headaches. On arrival to the emergency room, both patients presented with a high parasite load (12% vs. 4%). Infection was confirmed by blood smear and the tick panel showed a lack of coinfection in both cases.

The treatment of babesiosis depends on the clinical presentation and immune status of the patient. In asymptomatic individuals, the infection does not need to be treated. For those with mild disease, the 2006 Infectious Disease Society of America guidelines recommend atovaquone (750 mg per oral twice a day) and azithromycin (500-100 mg on day one and 250 mg per oral four times a day for seven to 10 days as first-line therapy [4,16]. For those with severe babesiosis, the Infectious Disease Society recommends intravenous clindamycin (300-600 mg every six hours) and quinine (650 mg every six-eight hours) [16]. However, based on studies published after 2006, UptoDate recommends intravenous azithromycin (500 mg four times a day) and per oral atovaquone (750 mg every 12 hours) [12]. This regimen is also associated with lower rates of adverse effects than the clindamycin and quinine regimen. The triple therapy of intravenous azithromycin, per oral atovaquone, and clindamycin can also be used [16]. For patients with severe disease, exchange transfusion can also be considered. Indications for the procedure include >10% parasite load, hemoglobin <10 g/dL, or pulmonary, liver, or renal impairment. Given the limited clinical data, the effectiveness and expected response to the above regimens is not well-characterized [4].

Both patients were started on the initial therapy of azithromycin 500 mg intravenous daily and atovaquone 750 mg per oral twice a day. They were also treated with doxycycline for a limited period of time for possible tick-borne coinfection. While the symptoms of the patient in Case 2 progressively improved within two days and his parasite load continually decreased, the Case 1 patient was not as responsive to the treatment regimen and required two exchange transfusions. 

Although the criteria for severe babesiosis is not clearly defined, both patients can be considered to have severe babesiosis based on their parasite load; however, the markedly higher parasitemia and severity of clinical presentation suggests that the first patient suffered from a more severe case. It is unclear why the infection presented with differing severity in the two patients. The more aggressive approach in the second patient with the azithromycin, atovaquone, and doxycycline regimen may have caused the milder presentation of the disease. Although the first patient’s age of 51 is a risk factor for severe disease (age >50 years old), the second patient is 48-years-old. Additionally, both patients reported tick bites from similar geographical locations and did not have a known immunocompromised state. Another possible explanation is the time between symptom onset and presentation. While the second patient presented after two days of symptoms, the first patient presented after approximately a week. 

Additionally, the above cases seem to suggest that the first-line therapy of antimicrobial agents may not be effective in cases of babesiosis with very high parasite loads. This is supported by the fact that the parasite load continued to increase dramatically in the first patient despite therapy. However, it is possible that the parasitemia would have declined on its own had the exchange transfusion been delayed. 

The above cases also suggest that for patients with a high parasite load, multiple exchange transfusion may be necessary for symptomatic resolution. This is in accordance with the American Society of Apheresis guidelines of exchange transfusions with the goal of <5% [17]. However, it is difficult to draw generalizable conclusions because of the small sample size.

As discussed above, babesiosis can cause severe symptoms. Preventive measures are very important in tick-borne infections such as Lyme disease, Anaplasma, and babesiosis. Protective clothing, tick repellents, and a timely visit to the doctor following a tick bite are important in preventing and controlling the disease [18-19].

## Conclusions

In clinical practice, babesiosis can present with varying severity depending on the length of tick attachment and the time of initiation of appropriate management. While patients with an earlier onset of a two-drug regimen therapy can achieve faster improvement of clinical symptoms, those with later initiation of therapy may require more aggressive and invasive ways of management to prevent the mortality and morbidity rate from the hemolytic anemia. As presented above in the two cases, the clinical presentation of the disease may vary significantly. The need for an exchange blood transfusion directly depends on the delayed seeking of medical therapy. While people living or traveling to the tick prominent parts of the country are aware of Lyme disease, they do not possess information about the possibility of getting babesiosis, which can lead to fatal consequences if not addressed in a timely manner. By educating the population about the clinical presentation and consequences of babesiosis, unwanted consequences may be significantly reduced.
